# Exhaust Emission Measurements from a Spark-Ignition Engine Using Fuels with Different Ethanol Content for Aircraft Applications

**DOI:** 10.1021/acsomega.2c02907

**Published:** 2022-08-17

**Authors:** Daniel Frank, Grace Neubauer, Christiane Bauer, Josef Kallo, Caroline Willich

**Affiliations:** † Institute for Energy Conversion and Storage, Universität Ulm, Albert-Einstein-Allee 47, 89081 Ulm, Germany

## Abstract

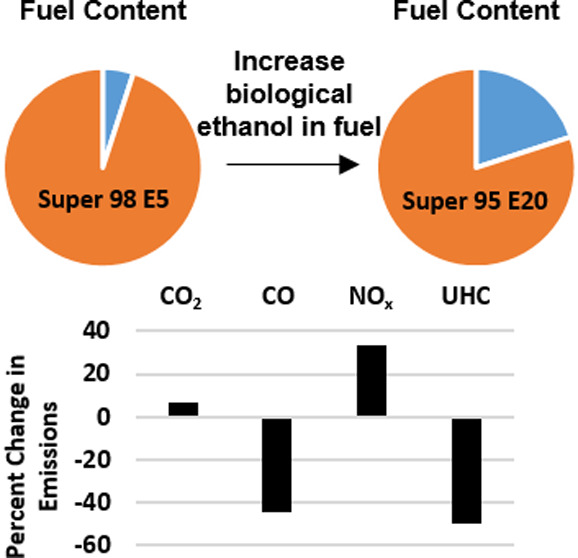

To have more sustainable aviation, ways to reduce lead and gaseous emissions are important and currently large research topics. As further efficiency improvements for internal combustion engines (ICE) have reached a limit, and the research and development of certifiable full and hybrid electric aircrafts are still ongoing, it becomes increasingly important to investigate the use of alternatives to conventional fuels, such as bioethanol. In this study, a state-of-the-art turbocharged 104 kW flight piston engine (BRP Rotax 915 iS) was tested with fuels containing various amounts of ethanol to assess the influence on the engine’s performance and emissions. Emission and performance maps covering the full range of engine operation from 4500 to 5800 RPM and about 40 to 110 kW output power were obtained using the standard fuel AvGas 100 LL, its current alternative Super 98 E5, and higher ethanol content fuels Super 98 E10 and Super 95 E20. With 20% ethanol in the fuel blend, a general decrease in CO and unburned hydrocarbon (UHC) emissions and an increase in CO_2_ and NO*_x_* were observed compared to the other fuels. Differences in the performance and emissions of the engine were also observed with different manifold air temperatures (MAT).

## 1 Introduction

To reduce the environmental impact of aviation while improving mobility, alternative fuels to replace fossil fuels, and especially leaded fuels that are still being used for single engine piston aircrafts (SEP), have been a topic of research for many decades now. There have been many efforts to phase out leaded fuels in small piston engine planes^1^ and replace them with nonleaded fuels, but particularly at secondary airports, low-leaded fuels are still in use. To completely phase out lead from spark-ignition internal combustion engines (SI ICEs) for SEP aircrafts, additional research is needed to find alternative additives as antiknocking agents in conventional aviation fuel or to find alternative fuels that can be used within aircraft piston engines. One possible alternative antiknock agent for use with unleaded gasoline is ethanol. It has been found that the research octane number (RON) as well as the engine thermal efficiency of gasoline can be increased with the addition of bioethanol and other oxygenated compounds.^2,3^

Lead is not the only engine exhaust compound that needs to be reduced. The reduction of climate-active gases like carbon dioxide (CO_2_), nitrogen oxides (NO*_x_*), carbon monoxide (CO), and unburned hydrocarbons (UHC) is also of interest in spark-ignition internal combustion engines (SI ICEs). So far, technological advancements, for example of the engine control system, have already resulted in reduced emissions.^4^ Although aircraft emissions are a small source of greenhouse gas emissions with respect to other industries,^4^ they are not negligible, and they also locally affect the area surrounding airports. One way of reducing the CO_2_ emissions in aviation is the increased use of biologically sourced fuels, such as ethanol. By utilizing ethanol fermented from biological sources, some degree of carbon neutrality can be achieved. Since the addition of ethanol to gasoline for usage in automotive engines is already a common practice, and automotive gasoline is less expensive than aviation gasoline (AvGas), utilizing automotive gasoline with ethanol added is a viable option for partially replacing fossil fuels.^5^

With the introduction of ethanol to the fuel, the heating value of the fuel decreases and so does the air-to-fuel ratio (AFR) for stoichiometric combustion. The stoichiometric AFR for 98.5% ethanol is 8.9 as compared to roughly 14.7 for pure gasoline,^6^ so ethanol requires less air for complete combustion since it is already being partially oxygenated. The latent heat of vaporization of ethanol, and other alcohols, is greater than that of gasoline. This lowers the temperatures in the cycle and contributes to a higher volumetric efficiency of the engine.^6^ Lower flame temperatures also lead to higher thermal efficiencies, even though the internal energy of the alcohol–air mixture is lower than that of gasoline and air.^6^ All of these factors relating to ethanol as the only fuel source are important to recognize, but as ethanol is added to gasoline mixtures, the properties of the mixture change. A disadvantage of bioethanol is that it can damage the fittings, sealings, and some metal components of the engine, and during colder months, cold start up of the engine is more difficult.^7,8^

Emission reduction from SI engines has already been reported in engines using ethanol-containing fuels. CO and UHC emissions have generally been found to reduce in concentration in new-generation SI engines with the addition of ethanol.^9^ Chen et al.^10^ found that for cold starting, an ethanol mixture between 20 and 30% decreased the UHC and CO emissions. Depending on the operating conditions of the tests, some studies report a decrease in NO*_x_* concentrations^11,12^ and some report an increase^13^ with increasing ethanol content in gasoline.^14^ Turner et al.^15^ found that NO*_x_* emissions decreased with the addition of ethanol but only until 85% ethanol by volume when testing at 1500 RPM and 3.4 bar indicated mean effective pressure (IMEP). Although the majority of research has found that there is an increase in CO_2_ emissions with the addition of anhydrous ethanol to gasoline as compared to pure gasoline,^16^ it can be compensated using biologically sourced ethanol for the aim of carbon neutrality.

For SI engines in general, there is a lot of research effort to reduce harmful emissions. Studies have been done to understand the in-cylinder processes and engine effects when using fuels containing ethanol from biological sources; however, there is a lack in experimental information of using these fuel varieties over the full operation range of SEP aircraft engines. The Swiss Federal Office of Civil Aviation (FOCA) database has been used to quantify the general landing and take-off cycle as well as cruise emissions from the SEP aircraft engines, including Lycoming as well as Rotax engines for compliance purposes. Although the database contains data on emissions for the Rotax 912, there is no current data on the Rotax 915, and the fuel used is generalized as a C_7_H_13_ MoGas, without even the addition of 10% ethanol.^17^ Similarly, the Airport Cooperative Research Program (ACRP) mentions that there is limited data on general aviation emissions as well, and performed their own emission measurements to perform statistical analysis with the FOCA and other data. Their findings resulted in some variance between the data sources, showing more information on GA emissions from piston engines are necessary for a comprehensive understanding of the emissions and their subsequent effect on the airport surrounding and atmosphere.^18^ This study also did not utilize alternative fuels for the characterization. Other studies performed tests for gaining a deeper understanding of the combustion processes with hydrous ethanol in a single piston test,^19^ emissions and combustion efficiency with n-butanol/kerosene in an SI aircraft engine to full load,^20^ and studies on general SI engines, with gasoline ethanol and acetylene fuel mixtures only at partial loads for engine combustion and emission analysis^21^ among many others. Still, the findings of higher than 10% ethanol–gasoline mixtures over the full range of aircraft engine operation are not completely understood. In this experimental study, a *Rotax 915 iS3* engine was tested with a variety of fuels to characterize the full emission, efficiency, and power maps to assess the impact of nonleaded and bioethanol-containing fuel usage in SEP aircraft engines on performance and emissions.

## 2 Materials and Methods (Experimental Setup, Model Description)

### 2.1 Testbench

To determine emissions and power, an engine testbench was set up. It contains a state-of-the-art 4-cylinder, 4-stroke turbocharged 104 kW flight piston engine (*BRP Rotax 915 iS*), an asynchronous load machine (*JS Technik Elektromotor-M3 315L-160 kW-4pol-B3*) with frequency inverter (*Toshiba VF-AS3*), a fresh air supply and exhaust gas removal system, a fuel supply system, and various cooling systems. The setup allows for the independent setting of manifold air pressure (MAP) and engine speed (RPM), within the respective operating range of the engine.

#### Cooling Systems

**Figure 1:**
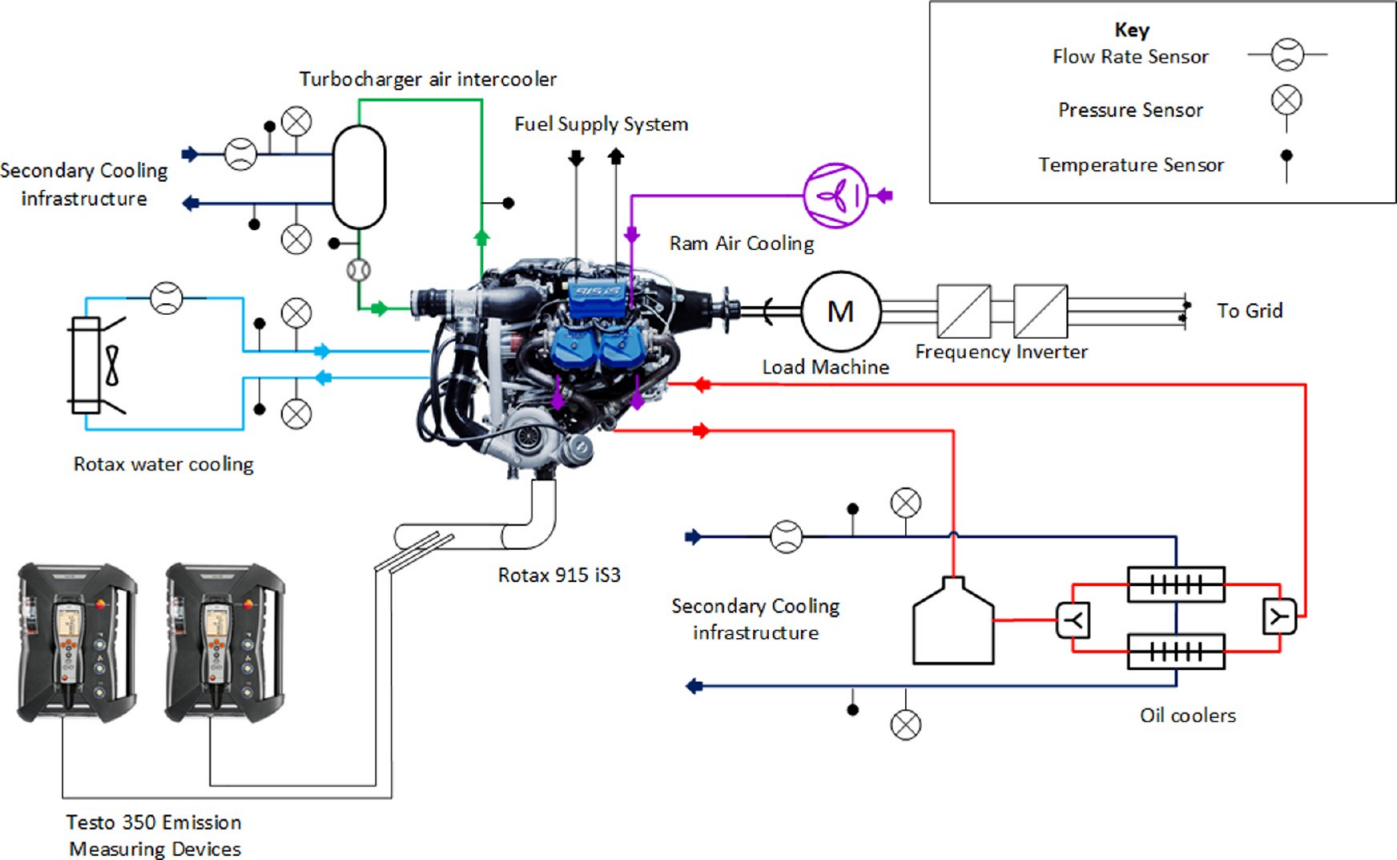
Engine testbench schematic. The engine is coupled to a load machine and a frequency inverter as a load. The charged air is cooled with an air-to-water intercooler, the oil with oil-to-water heat exchangers, the cylinder walls with ram air cooling, and the cylinder heads with water cooling. The emission measuring systems are connected with hoses to probes attached to the exhaust gas pipe of the engine. Photographs of engine and devices courtesy of BRP Rotax^22^ and Testo.^23^ Copyright 2022.

The testbench schematic and cooling systems can be seen in Figure 1. The water-cooling circuit of the *Rotax 915* engine, which mainly cools the cylinder heads, is set up as an independent circuit driven by the internal water pump of the engine. The heat is removed via a water-to-air finned heat exchanger with electric fans to the ambient air. The coolant is a 50/50 by volume water–ethylene glycol mixture. Sensors are installed to measure the mass flow rate (*ifm SV7050*), pressure drop (*ifm PT5504*), and inlet and outlet temperatures (*ifm TM4101 Pt100 and ifm SV7050*) over the heat exchanger. A thermostat (*Franz Aircraft thermostat-kit 10807*) is located at the inlet of the engine.

The cylinder walls are equipped with cooling fins. The ram air used for the forced convection over the fins is provided by an *ebmpapst G2E140 radial* blower.

The Rotax oil cooling circuit is connected to the lab cooling infrastructure by two oil-to-water coolers (*Laminova C43-182)* through which the oil flows in parallel while the water is in series. The oil temperature is measured by the engine control unit (ECU), and sensors are installed to measure the coolant mass flow rate (*ifm SV7050*), pressure drop (*ifm PT5504*), and inlet and outlet temperatures (*ifm TM4101 Pt100 and ifm SV7050*) of the coolers. For cooling the charged air from the turbocharger, an air-to-water intercooler (*PWR PWI2292*) is utilized before the air enters into the manifold. At the intercooler, inlet and outlet air temperatures are measured (*Type K thermocouples*), whereas the ECU measures the MAP, the manifold air temperature (MAT), and the air mass flow rate (AF). On the coolant side, the pressure drop, inlet and outlet temperatures (*ifm PT5504 and ifm TM4101*), and mass flow rates (*ifm SV7150*) are measured. The exhaust gas cooling system uses two air-to-water stainless steel exhaust gas heat exchangers in series. Here, the coolant flow rate (*ifm SV5150)*, in- and outlet pressure (*ifm PT5504)*, and temperatures (*ifm TM4101* and *ifm SV5150*) are measured. The secondary cooling infrastructure is designed to remove 100 kW of heat. The data from the sensors are logged by a data logger (*Keysight LXI 34972A*).

#### Fuel Supply System

For safety reasons, the fuel is stored in a stainless steel fuel tank within a safety cabinet. Using a fuel pump (*Rotax Kraftstoffpumpe ID 889697*), the fuel is transported to the engine.

#### Control

Engine speed and MAP are set by the operator. The engine speed, respectively, the inverter frequency is set via a CANopen network. The MAP is set by adjusting the throttle position via an electrical linear actuator (*Actuonix L16-100-35-12-P*).

### 2.2 Sensors and Emission Compounds

The exhaust emissions from the *Rotax 915* engine were measured by a *Testo 350* emission measuring system. The emission measuring devices used flue gas coolers for condensing the water and conditioning the dry flue gas to a temperature of 3 °C. The details, as well as accuracy and measurement limitations, of the *Testo 350* measuring system are given in Table 1.

**Table 1:** Testo350 Sensor Characteristics

compound	sensor type	detection limit^24^	accuracy^24^	dilution
CO	electrochemical	0 ppm	±2% and ±10 ppm (0...199 ppm) ±5% of reading (200...2000 ppm) ±10% of reading (rest of range)	40×
NO_2_	electrochemical	0 ppm	±5% and ±5 ppm (0...99.9 ppm) ±5% of reading (rest of range)	5×
NO	electrochemical	0 ppm	±5% and ±5 ppm (0...99 ppm) ±5% of reading (100...1999.9 ppm) ±10% of reading (rest of range)	5×
SO_2_	electrochemical	0 ppm	±5% and ±5 ppm (0...99 ppm) ±5% of reading (100...1999 ppm) ±10% of reading (rest of range)	5×
O_2_	electrochemical	0%	±0.2 vol %	0×
CO_2_	infrared	0%	±0.3 vol % ±1% of reading	0×
UHC	heat effect/combustion	250 ppm	±5% and ±400 ppm (100...4000 ppm) ±10% of reading (rest of range)	5×

The cross sensitivities of the sensors also were accounted for, in which only UHC had a 35% cross sensitivity with incoming CO gas, and NO_2_ had less than -2% cross sensitivity with SO_2_.^24^ To detect all species within the measuring ranges and to account for cross sensitivities, the emission measuring system consists of two devices. One device has sensors for CO_2_ and O_2_ (all undiluted), as well as CO (diluted by 40×), while the other device has NO, NO_2_, SO_2_ (only used with AvGas 100 LL), and UHC (all diluted by 5×).

### 2.3 Fuels

The relevant information on the fuels used in the experiments is found in Table 2. These fuels were chosen to compare the low-lead aviation fuel to the nonleaded alternatives and fuels with biological sourced content. With the increase in ethanol in the fuel mixture, the stoichiometric AFR also changes. For Super 98 E5, it is 14.42, and for Super 95 E20, it is 13.56.^25^

**Table 2:** Fuel Properties^a^

fuel type	RON	specification	LHV (MJ/kg)	ethanol content up to (vol %)
Super 98 E5	min. 98^26^	DIN EN 228	41.99^27^	5
Super 95 E10	min. 95^26^	DIN EN 228	41.24^27^	10
Super 95 E20	min. 85^26^	DIN EN 228	40.967^27^	20
AvGas 100 LL	MON: min. 99.6^28^	ASTM D 910	43.5^28^	ca. 0

a AvGas 100 LL, Super 98 E5, and Super 95 E10 were purchased as-is, and Super 95 E20 was made in-house by mixing Super 95 E10 and 96% bioethanol with the splash blending method.

### 2.4 Procedure

During the commissioning phase of the setup, preliminary measurements were conducted. The preliminary data indicated a stabilization time for the emissions of up to 5 minutes after the transition to a given setpoint (RPM, MAP). For the stability of the emissions, thermal stability is a prerequisite. Therefore, to ensure reliable data, after the engine start and warm-up phase according to the operator’s manual,^29^ the engine was preconditioned at an application relevant temperature level for the needed thermal stability for the emission measurements. For this, the engine was set at 4500 RPM and MAP ≈ 86 kPa for 6 min resulting in an output power of 45 ± 1 kW for all tested fuels. During the measuring phase, the setpoints were performed each for 6 min (Table 3, except 5800 RPM setpoints).

**Table 3:** Rotax Testing Setpoints

RPM	MAP [kPa or throttle %]					
4500	86	102	119	135	152	100% (WOT)
5000	86	102	119	135	152	100% (WOT)
5500	86	102	119	135	152	100% (WOT)
5800	98%					100% (WOT)

As can be seen in Table 3, six different throttle positions were performed for each RPM, whenever possible. The last setpoint is a wide open throttle (WOT).

The 5800 RPM setpoints are not considered as continuous operating setpoints since the maximum allowed duration for engine speed over 5500 RPM is 5 min.^29^ In airplane operations, these setpoints are typically only used in the start and climb phase at the beginning of the flight. Therefore, an airplane take-off and climb envelope was simulated to measure the emissions.

Depending on the linearized throttle position, the ECU sets the control mode to economy or power mode. While in economy mode, the mixtures tend to be close to stoichiometric or lean, in power mode, the mixtures are richer as indicated by the AFR. To evaluate the difference between maximum power as well as emissions in economy mode (98% throttle) and power mode (WOT), the following procedure was implemented:

1. Engine start up to 1800 RPM at approximately 35% throttle.
2. Warm-up phase at 2000 RPM and approximately 44% throttle for 2 min.
3. Warm-up phase at 2500 RPM until the oil temperature is 60 °C.
4. Taxi simulation at 2000 RPM for 1 min.
5. Take-off simulation at 5800 RPM and appropriate throttle position for 5 min.

### 2.5 Calculation Methods

#### 2.5.1 Cross-Sensitivity

To account for the cross sensitivities that occur in the sensors from the exhaust mixture, adjusted concentrations are calculated as given by the Testo 350. For example, 35% of the CO measurement must be subtracted from the UHC measurement to account for this cross sensitivity. Equation 1 displays the calculation of *X*_adjusted_ where *X*_displayed_ is the concentration from the sensor reading, *Y*_displayed_ is the cross-gas concentration reading, and *Z* is the decimal value by which to adjust.
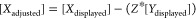
1

#### 2.5.2 Efficiency

The engine efficiency is calculated as follows according to eq 2.
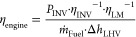
2

*P*_INV_ is the measured output power of the frequency inverter, *ṁ*_Fuel_ is the measured fuel mass flow rate, and Δ*h*_LHV_ is the lower heating value of the given fuel. The efficiencies of the inverter and the load machine are η_INV_ = 0.98 and η_LM_ = 0.958, respectively.^30,31^

## 3 Results and Discussion

The measured engine performance and exhaust emission concentrations are given in the following section. As described in the Materials and Methods section, the concentrations are given as “dry” concentrations since they have been cooled and the humidity in the flue gas removed, meaning that the shown concentrations will be higher than the same case with humid gas.

### 3.1 Engine Performance

**Figure 2:**
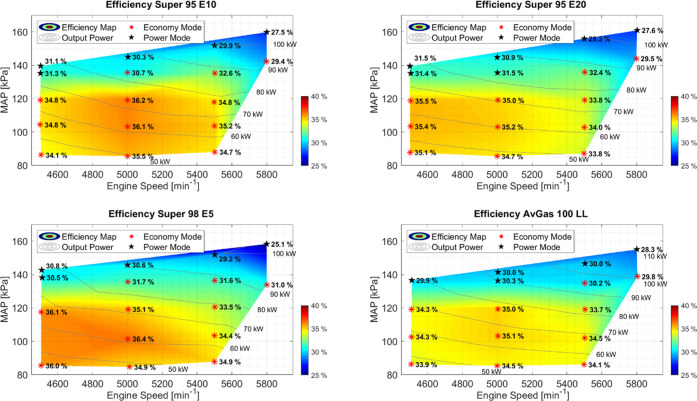
Engine efficiency maps for different fuels at different engine speeds, MAP, and powers. The range of efficiencies over the entire operating range was measured. From bottom right clockwise to top right, the fuels tested were AvGas 100 LL, Super 98 E5, Super 95 E10, and Super 95 E20. Setpoints in “Power mode” are marked with a black star.

The measurement campaign was conducted under ambient conditions, and the output power as well as the fuel flow and the chemical emissions were measured with the previously described method (eq 2). The maximum power reached for the different fuels was 109.6 kW for Super 98 E5 at 43.9 °C MAT, 109.2 kW for Super 95 E10 at 43.6 °C MAT, 111.8 kW for Super 95 E20 at 39.2 °C MAT, and 110.7 kW for AvGas 100 LL at 41.6 °C MAT. For each setpoint, the efficiency was calculated. Figure 2 shows the results of efficiency over engine speed and MAP for the different fuels. Between the measured points, a piecewise linear interpolation was used. For better comparison, the figures share a common color scale.

The tests with AvGas 100 LL as fuel resulted in a MAT range of 23.5 to 41.6 °C. As seen in Figure 2, the efficiency ranged from 28.3 to 35.1% with the minimum efficiency at the setpoint with the highest power and the maximum efficiency in the middle area of the efficiency map. The efficiencies decreased significantly with increasing power, especially for those setpoints where the ECU had switched the control strategy to power mode.

The tests performed with Super 98 E5 as fuel resulted in a MAT range of 39.5 to 56.2 °C. In this case, the efficiency ranged from 25.1 to 36.4%. The overall pattern of the efficiency map matches the pattern of the efficiency map for AvGas 100 LL with the minimum efficiency at the setpoint with the highest power and the maximum efficiency in the middle area of the efficiency map. The minimum efficiency of 25.1% was obtained at far-from-optimal conditions, as the engine log files indicated knocking events at this setpoint.

When Super 95 E10 was used as fuel, the MAT ranged between 29.1 and 55.4 °C. The general aspect of the efficiency maps is similar to those of the efficiency maps of AvGas 100 LL and Super 98 E5 with peak efficiency (36.2%) in the middle of the map and significantly decreasing efficiencies for higher powers, with the minimum efficiency of 27.5% at maximum power.

The measurements with Super 95 E20 as fuel resulted in a MAT ranging between 28.6 and 60.1 °C. Differing from the AvGas 100 LL, Super 98 E5, and most interestingly Super 95 E10 measurements, the highest efficiency of 35.5% was observed at lower engine speed at the left border of the measured map. Decreasing efficiency with increasing power as well as power mode setpoints was also observed for Super 95 E20 as for the other fuels, and the minimum efficiency of 27.6% was observed at maximum power.

### 3.2 Exhaust Gas Temperatures

**Figure 3:**
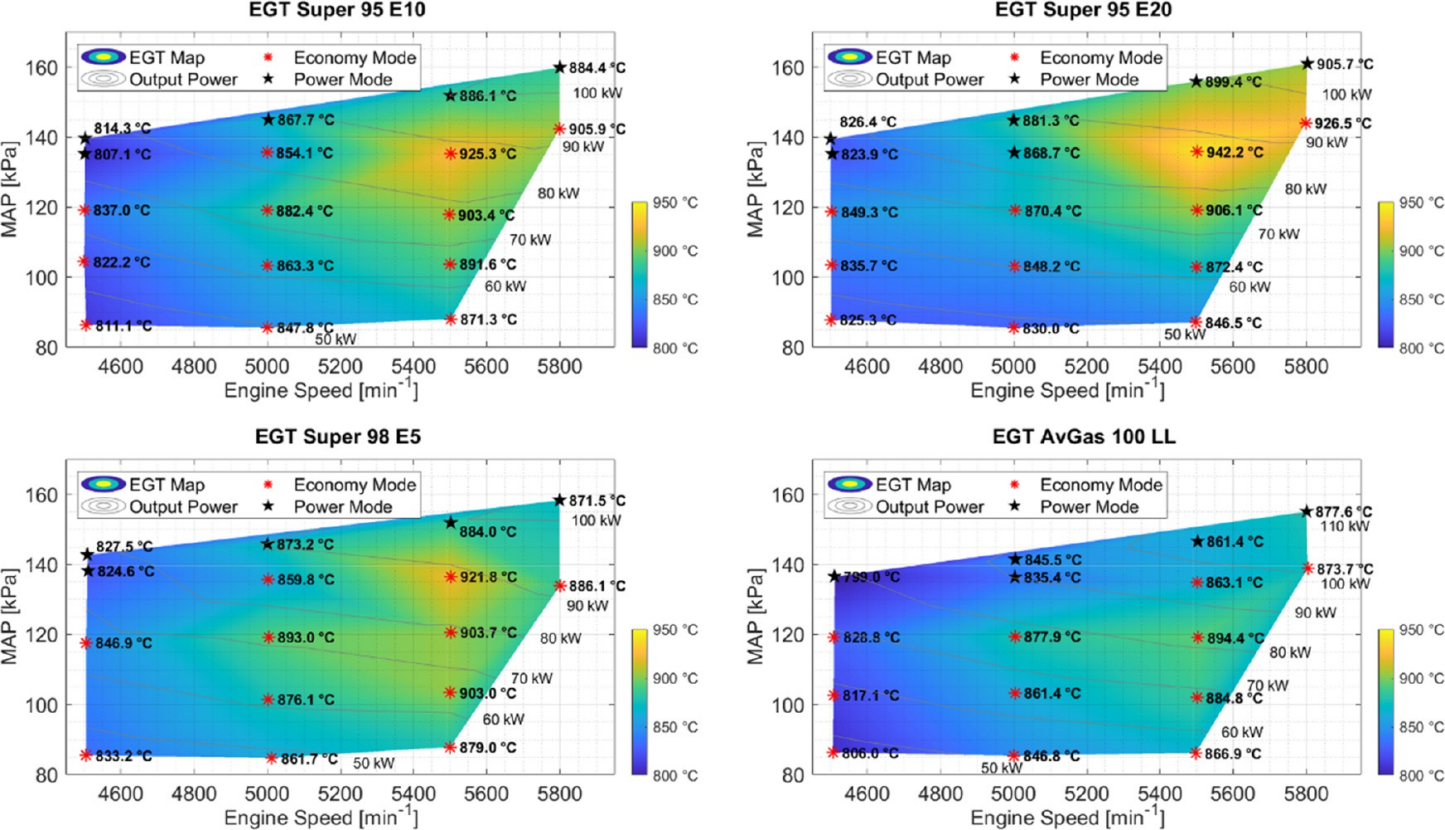
Engine exhaust gas temperature maps for different fuels at different engine speeds, MAP, and powers. The range of average EGTs over the entire operating range was measured. From bottom right clockwise to top right, the fuels tested were AvGas 100 LL, Super 98 E5, Super 95 E10, and Super 95 E20. Setpoints in Power mode are marked with a black star.

The average EGTs for each fuel tested are given in Figure 3. The maximum EGT of each fuel is observed at an engine speed of 5500 rpm and an output power in the range of 80–90 kW. Overall, the maximum EGT is observed with Super 95 E20, whereas for AvGas 100 LL, the lowest maximum temperature was observed. What is also seen is that the EGT temperature span is larger for Super E10 and Super E20, where the range is over 110 K difference, whereas for AvGas 100 LL, the span is under 90 K difference.

### 3.3 Unburned Hydrocarbons

**Figure 4:**
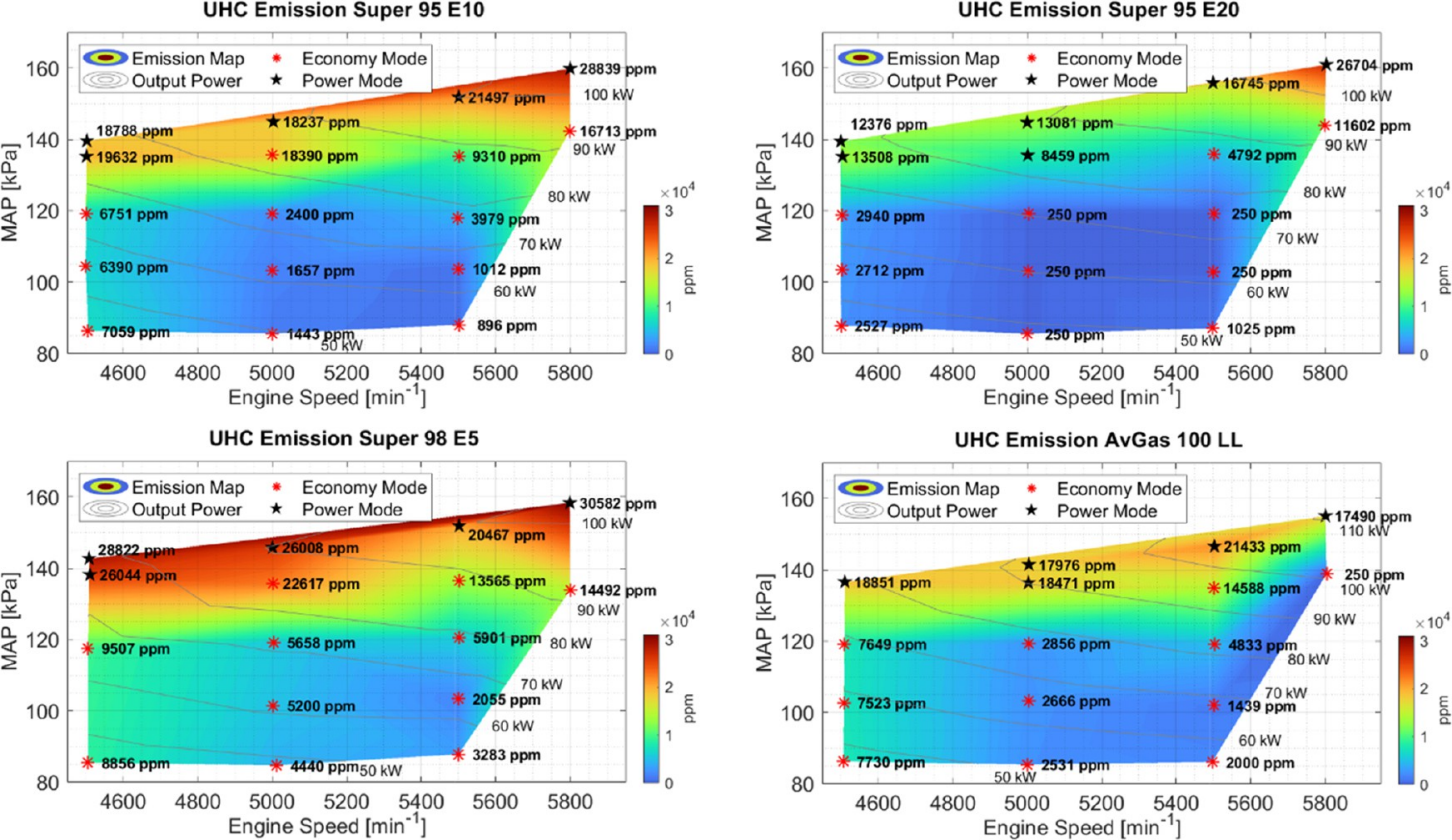
Concentration of unburned hydrocarbons for each individual fuel type at different engine speeds, MAP, and power. The measured concentrations over the operating range are plotted. From bottom right clockwise to top right, the fuels tested were AvGas 100 LL, Super 98 E5, Super 95 E10, and Super 95 E20. Setpoints in Power mode are marked with a black star.

The unburned or partially burned hydrocarbon concentration maps are given in Figure 4. It can be seen that for all fuels, the concentration of UHC in the exhaust gas increased with increasing power. These higher concentrations are in part a consequence due to the change in the control strategy for these setpoints, where the engine is in power mode. For Super 95 E20, several setpoints had UHC concentrations under the detection limit of the UHC sensor in the *Testo 350* (250 ppm). This was the case for midrange RPM (5000–5500 RPM) and lower MAP (85 kPa to 119 kPa) setpoints. A general trend seen for the Super fuels at all setpoints was that with increasing ethanol content, the concentration of UHC decreased. This decrease in overall UHC concentrations reflects a more complete combustion. With higher ethanol content in the fuel, the overall average atomic weight of the fuel is lower. As the average length of carbon chains in the fuel decreases, the combustion of hydrocarbons is more complete. The average EGT ranges that were measured for each fuel were: AvGas 100 LL (799–894 °C), Super 98 E5 (827–922 °C), Super 98 E10 (807–925 °C), and Super 95 E20 (825–942 °C), which also indicates a more complete combustion for higher ethanol contents.

The measured UHC concentrations were lower in general for AvGas 100 LL than Super 98 E5.

### 3.4 Nitrogen Oxides

**Figure 5:**
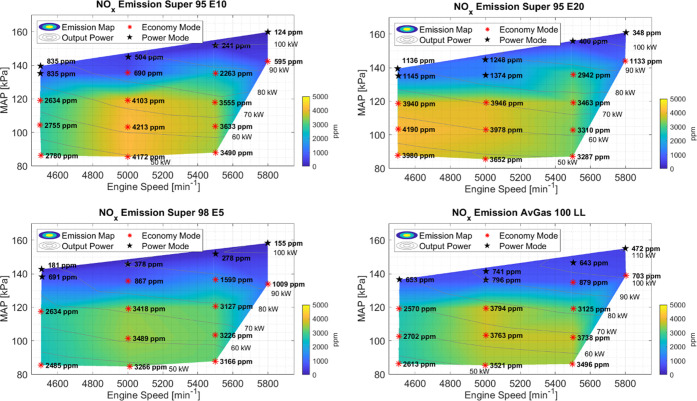
Concentration of NO*_x_* for each individual fuel type at different engine speeds, MAP, and power. The measured concentrations over the operating range are plotted. From bottom right clockwise to top right, the fuels tested were AvGas 100 LL, Super 98 E5, Super 95 E10, and Super 95 E20. Setpoints in Power mode are marked with a black star.

The NO*_x_* emissions for all fuels can be seen in Figure 5. As expected, the NO*_x_* concentration was higher for stoichiometric and lean mixtures, and the highest concentrations of NO*_x_* were obtained at midrange power. The lowest NO*_x_* concentrations are seen for richer AFRs during power mode. Also visible from Figure 5 is the increase in NO*_x_* with an increasing ethanol content of the Super fuels, with Super 98 E5 having the overall lowest NO*_x_* emissions of all four fuels. The increasing NO*_x_* concentrations can be partially explained by the increase in temperature, as the EGT ranges increased slightly, as seen in Section 3.2 and Figure 3, where Super E10 and Super E20 have the highest EGTs as compared to Super E5 and AvGas 100 LL. Important to note is that the highest EGTs do not necessarily correlate with the highest NO*_x_* concentration, showing that AFR is a significant factor in NO*_x_* formation. In general, the likeliness of forming nitrogen oxide radicals increases with temperature, although it is also influenced by other parameters such as AFR.

### 3.5 Carbon Dioxide

**Figure 6:**
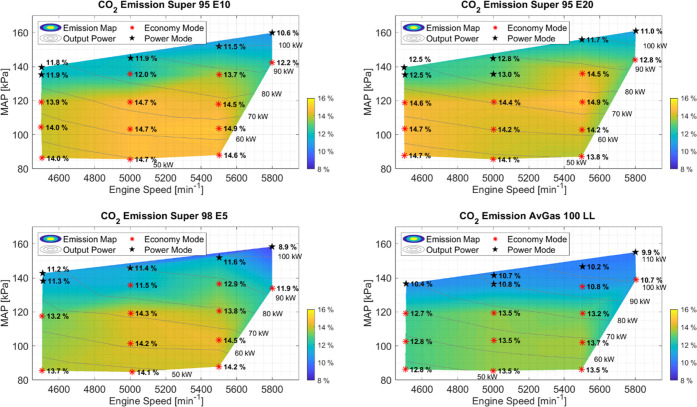
Concentration of CO_2_ for each individual fuel type at different engine speeds, MAP, and power. The measured concentrations over the operating range are plotted. From bottom right clockwise to top right, the fuels tested were AvGas 100 LL, Super 98 E5, Super 95 E10, and Super 95 E20. Setpoints in Power mode are marked with a black star.

Figure 6 shows the CO_2_ emissions measured for each fuel. For AvGas 100 LL, it is seen that for most setpoints, the CO_2_ concentration is lower than the other fuels. Observing the Super fuels with increasing ethanol content, a higher concentration of CO_2_ is seen. With increasing MAP, the CO_2_ emissions generally decreased, and CO_2_ emissions were higher for setpoints with high engine efficiency at midrange power. At the high power setpoints, the lowest CO_2_ concentrations were measured. Due to the increase in the fuel injection rate at 5800 RPM and WOT, a richer mixture is used leading to a decrease in the completeness of combustion for the high power setpoints. The CO_2_ emissions measured for the different fuels are also an indicator of how the completeness of combustion is influenced by the ethanol at particular setpoints of the engine. As previously explained for UHC, the increase in ethanol decreases the average molar mass of the fuel, leading to more complete combustion which is reflected in this increase in CO_2_ emissions. Although the CO_2_ does increase with increasing ethanol, UHC decreases, reducing the adverse effects of UHC on the environment.

### 3.6 Carbon Monoxide

**Figure 7:**
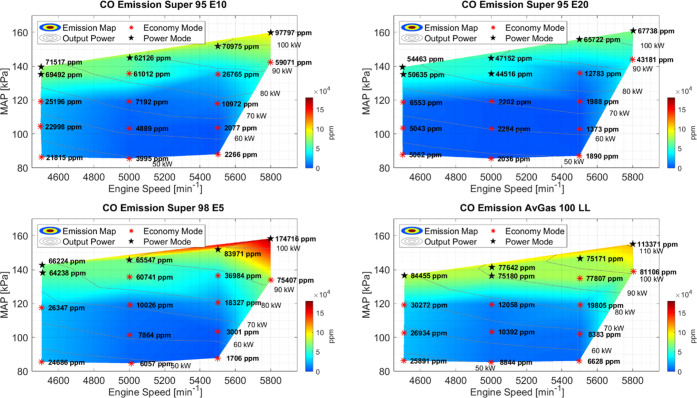
Concentration of CO with respect to the fraction of available power for each individual fuel type. The measured concentrations over the operating range are plotted. From bottom right clockwise to top right, the fuels tested were AvGas 100 LL, Super 98 E5, Super 95 E10, and Super 95 E20. Setpoints in Power mode are marked with a black star.

The CO measurements can be seen in Figure 7. CO is an important emission to examine because of its lethal nature in high quantities and its tendency to oxidize to CO_2_ over time in the presence of oxygen. At 5800 RPM and WOT, the CO emissions are highest for Super 98 E5 and the lowest for Super 95 E20. At 5800 RPM and WOT for Super 98 E5, there were also engine knocking events that were not present for all of the rest of the setpoints and fuels, which can also contribute to such a high CO concentration. As the power decreased, the general trend for all fuels is that the CO concentration decreases as well. Super 95 E20 has the least CO emissions of all tested fuels. Since CO is an incomplete combustion product, this corresponds to the higher CO_2_ concentrations and more complete combustion with this fuel. When CO decreases, CO_2_ values increase and vice versa. Because the oxygen content per gram of Super E20 is higher than Super E5, the combustion efficiency increases and the stoichiometric AFR decreases.^9^ This then results in the AFR, according to the chosen setpoints, being leaner, in which the combustion is more complete at these points resulting in lower CO emissions.^32^ Increasing the ethanol amount in the fuel can help reduce CO emissions.

### 3.7 Temperature Dependence (MAT)

Several setpoints for Super 98 E5, Super 98 E10, and Super 95 E20 were tested in cold (MAT range 32.0 to 43.9 °C) and hot (MAT range 43 to 56.8 °C) conditions. Those setpoints are shown in Table 4.

**Table 4:** Setpoints Tested for Hot and Cold Conditions

setpoint	engine speed	MAP or throttle	ECU mode
1	5500 RPM	119 kPa	economy mode
2	5000 RPM	WOT	power mode
3	5800 RPM	WOT	power mode

**Figure 8:**
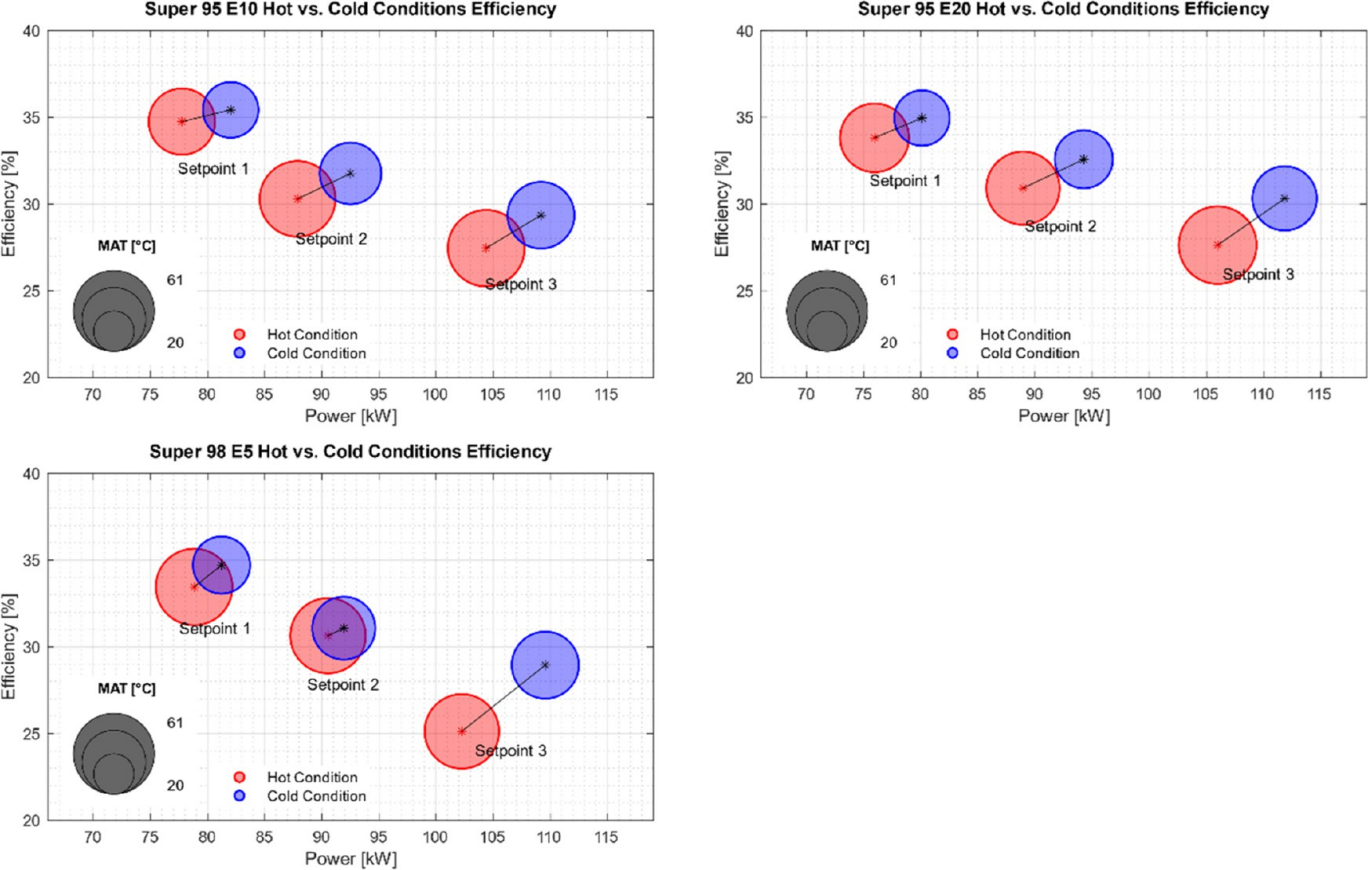
Engine efficiency under hot and cold manifold air temperature conditions. The size of the marks corresponds to the MAT, and the red marks correspond to hot condition and blue to cold.

With decreasing MAT, an increase in the output power and efficiency was observed. The shift in power and efficiency can be seen in Figure 8, where the size of the circles corresponds to the MAT. For the cold condition points, the temperature spread between MAT and EGT was at least 20 K larger than for the hot condition points. The greater temperature spread between MAT and EGT indicates a higher thermodynamic efficiency, which results in an increased overall efficiency, as shown in Figure 8.

**Figure 9:**
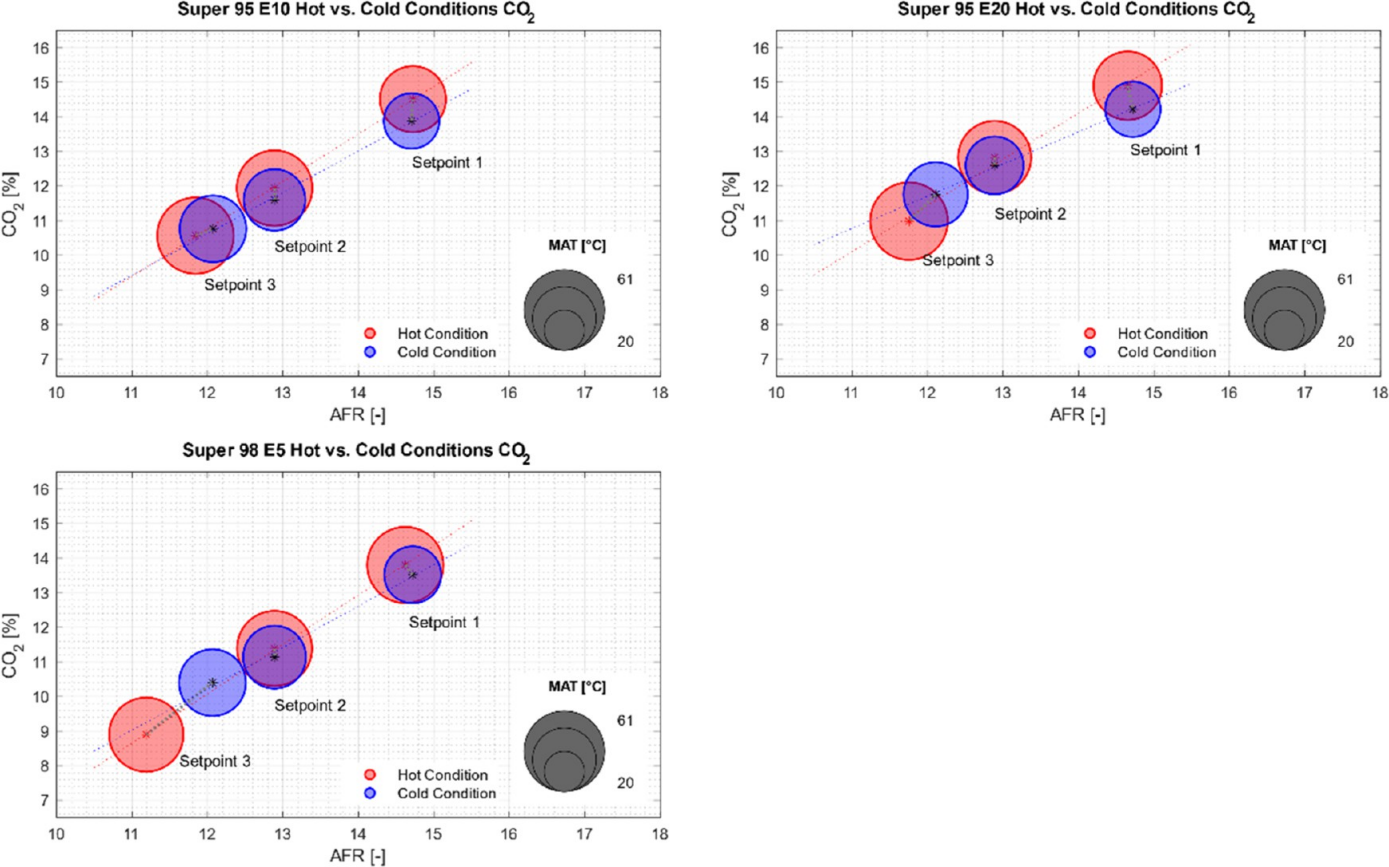
CO_2_ emissions under hot and cold manifold air temperature conditions. The size of the marks corresponds to the MAT, and the red marks correspond to hot condition and blue to cold. The slope of the hot condition and cold condition points are also plotted.

The MAT also influenced the emissions. In Figure 9, the CO_2_ emissions vs AFR for the hot and cold conditions are shown. In general, increasing CO_2_ emissions for increasing AFR are observed for all tested fuels. When analyzing the temperature regimes individually (hot and cold conditions) for the measured setpoints, a linear dependency between AFR and CO_2_ emissions is observed for all fuels, where the slope depends on the MAT regime. The AFR ranged from rich to stoichiometric for Super 98 E5 and from rich to lean for Super 95 E10 and Super 95 E20. Since the engine was not tested at higher AFR, the relationship between CO_2_ and AFR may not be valid at even leaner conditions. Both setpoints 1 and 2 show lower CO_2_ emissions for the cold conditions in comparison to the hot conditions for all fuels. Apart from the variation in temperature, the external conditions for the combustion (e.g., available time as a function of RPM) stay the same for every setpoint. Lower temperatures result in a lower probability of CO_2_ formation and therefore in the observed reduction in CO_2_ emissions. For setpoint 3, an increase in CO_2_ emissions is observed with lower temperatures. In this case, the increase with the change toward leaner AFR appears to be the dominant effect.

## 4 Conclusions

The engine performance and the resulting emissions (CO_2_, CO, NO*_x_*, and UHC concentrations) in a *Rotax 915* were examined for various fuels containing different amounts of ethanol. Ethanol-containing fuels were Super 98 E5, Super 98 E10, and Super 95 E20. AvGas 100 LL was also measured for comparison. The tests were performed in a laboratory setting and over the entire operating range of the engine.

The maximum power reached for the different fuels was 109.6 kW for Super 98 E5 at 43.9 °C manifold air temperature (MAT), 109.2 kW for Super 95 E10 at 43.6 °C MAT, 111.8 kW for Super 95 E20 at 39.2 °C MAT, and 110.7 kW for AvGas 100 LL at 41.6 °C MAT, while the nominal performance is 104 kW. For all fuels, the overall efficiencies decreased significantly with increasing power. The unburned hydrocarbon (UHC) and CO concentrations generally increased with the manifold air pressure, while CO_2_ concentrations in the exhaust for these same setpoints decreased for all fuels. The highest NO*_x_* concentrations were found for stoichiometric and lean mixtures, which occurred at midrange power. When increasing the amount of ethanol in the fuel, the UHC and CO concentrations in the exhaust decreased, while higher NO*_x_* and CO_2_ concentrations were measured for the higher ethanol content. The measurements showed that higher ethanol content in gasoline for use in aviation (e.g., Super 95 E20) has the potential to decrease certain engine emissions, especially UHC and CO, without a significant effect on power or efficiency. Super 95 E20 caused an increase in CO_2_ emissions, but with ethanol produced from renewable sources, more carbon neutrality can be achieved in aviation.

In this study, the engine was used off-the-shelf, without any adjustments to the engine control. To further optimize performance and emissions, modifications to the engine and engine control can be considered. The measured CO and UHC concentrations decreased with higher ethanol content but NO*_x_* increased. Adjusting the air–fuel ratio to richer mixtures could result in lower NO*_x_* concentrations for these fuels.

For using ethanol blends with gasoline in airplanes, other aspects need to be considered as well since they might limit the utilization of high ethanol content fuels in flight. For example, ethanol-containing fuels might increase the risk for phase separation and potential vapor lock.^5^ This might result in a reduced service ceiling for the aircraft. Implementing a modified version of a multi-point fuel injection (MPFI) with separate storage and injection of a low-ethanol content fuel and ethanol is one possible technical approach to mitigate this constraint. The mixture, that in this study has been supplied to the engine as one single fuel, would be created within the cylinder by separate injection of fuel and ethanol. In case of vapor lock in the ethanol injection system, the engine could still be operated using only the low-ethanol content fuel, mitigating the beforehand mentioned reduction in the service ceiling.

The experiments showed that the use of higher ethanol content fuels for aviation is feasible in terms of engine performance and emissions, but further research and development are required for its actual usage in flight.

## Abbreviations Used

[X]: concentration of a given compound X (ppm or %)
AF: air mass flow rate (g min^–1^)
AFR: air-to-fuel ratio
AvGas 100 LL: aviation gasoline with 100 MON octane rating, low lead
CO: carbon monoxide
CO_2_: carbon dioxide
ECU: engine control unit
EGT: exhaust gas temperature (°C)
E5, E10, E20: ethanol content up to 5, 10, 20%
ICE: internal combustion engine
IMEP: indicated mean effective pressure (bar)
MAP: manifold air pressure (kPa)
MAT: manifold air temperature (°C)
MPFI: multipoint fuel injection
NO: nitric oxide
NO_2_: nitrogen dioxide
NO*_x_*: nitrogen oxides
O_2_: molecular oxygen
SEP: single engine piston
SI: spark-ignition
SO_2_: sulfur dioxide
Super 95, 98: motor gasoline with 95, 98 RON octane rating
UHC: unburned hydrocarbons
WOT: wide open throttle
RPM: revolutions per minute (min^–1^)
